# Absence Seizure Control by a Brain Computer Interface

**DOI:** 10.1038/s41598-017-02626-y

**Published:** 2017-05-29

**Authors:** Vladimir A. Maksimenko, Sabrina van Heukelum, Vladimir V. Makarov, Janita Kelderhuis, Annika Lüttjohann, Alexey A. Koronovskii, Alexander E. Hramov, Gilles van Luijtelaar

**Affiliations:** 1 REC ‘Nonlinear Dynamics of Complex Systems’, Yuri Gagarin State Technical University of Saratov, Polytechnicheskaya 77, Saratov, 410054 Russia; 2 Institute of Physiology I, University of Münster, Robert-Koch-Str 27a, 48149 Münster, Germany; 3 Saratov State University, Astrahanskaja 83, Saratov, 410012 Russia; 4 Donders Centre for Cognition, Radboud University, Nijmegen, The Netherlands

## Abstract

The ultimate goal of epileptology is the complete abolishment of epileptic seizures. This might be achieved by a system that predicts seizure onset combined with a system that interferes with the process that leads to the onset of a seizure. Seizure prediction remains, as of yet, unresolved in absence-epilepsy, due to the sudden onset of seizures. We have developed a real-time absence seizure prediction algorithm, evaluated it and implemented it in an on-line, closed-loop brain stimulation system designed to prevent the spike-wave-discharges (SWDs), typical for absence epilepsy, in a genetic rat model. The algorithm corretly predicted 88% of the SWDs while the remaining were quickly detected. A high number of false-positive detections occurred mainly during light slow-wave-sleep. Inclusion of criteria to prevent false-positives greatly reduced the false alarm rate but decreased the sensitivity of the algoritm. Implementation of the latter version into a closed-loop brain-stimulation-system resulted in a 72% decrease in seizure activity. In contrast to long standing beliefs that SWDs are unpredictable, these results demonstrate that they can be predicted and that the development of closed-loop seizure prediction and prevention systems is a feasable step towards interventions to attain control and freedom from epileptic seizures.

## Introduction

Bilateral generalized spike and wave discharges (SWDs), the electrophysiological hallmark of absence epilepsy, are generated within the cortico-thalamo-cortical network^1–3^. It has long been thought that SWDs are unpredictable and suddenly arise from a normal background EEG^4^. Offline analysis of available EEG data sets with advanced network analysis techniques however, have revealed intracortical and cortico-thalamo-cortical changes in network activity that could be detected as early as 2 seconds before SWD onset^5, 6^. SWDs are also preceeded by short lasting delta-theta precursors in the cortex and thalamus^7^. In the current paper we present a new SWD/absence seizure prediction algorithm, which assesses in real-time the changes in synchrony between cortex and thalamus that predict SWDs.

**Figure 1:**
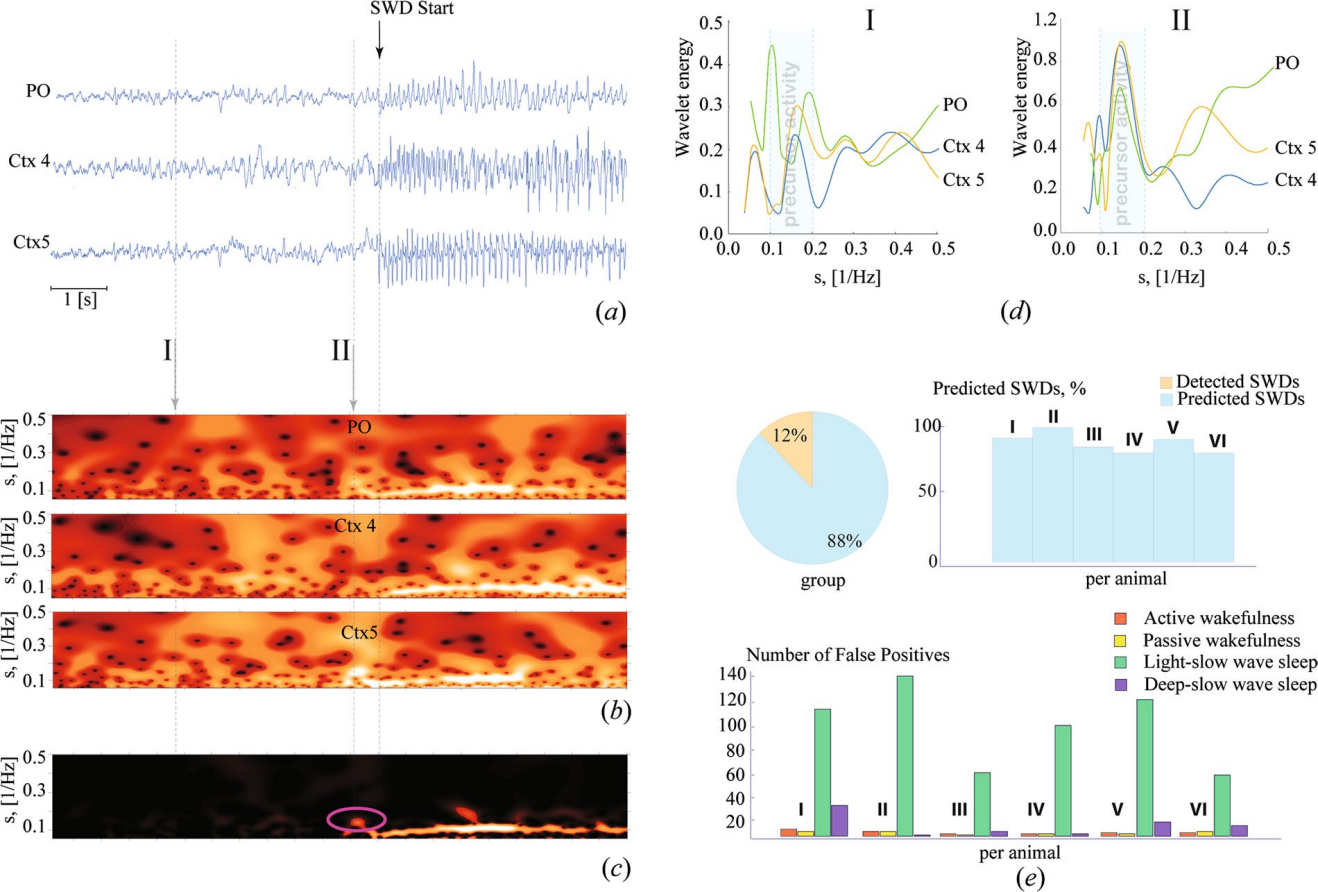
(**a**) A set of EEG recordings taken from subgranular layers 4 (Ctx4) and 5 (Ctx5) of the somatosensory cortex and postero/lateral nucleus of the thalamus (PO). (**b**) Energy of wavelet transformation, corresponding to the EEG signals shown above and distributed over the range of timescales *s* = 1/*f*, where *f* is the linear frequency. (**c**) The resulting surface $W(s,t)={\prod }^{}{W}_{i}(s,t)$. The oscillatory pattern occurring prior to SWD onset, which is considered as a precursor of SWD, is encircled. (**d**) The momentary distributions of the wavelet energy in the 5–10 Hz band, taken for 4 seconds (I) and ~0.5 second (II) before SWD onset. The left figure shows that at 4 seconds before SWD onset only cortical EEGs exhibit a local synchronization in the precursor band (i.e. the local increase of energy appears in the frequency ~7 Hz). The wavelet energies of the cortical EEGs exhibit a synchronized increase (i.e. the lines are aligned to each other), while the wavelet energy of the thalamic EEG does not (i.e. the thalamic line is shifted relative to the others). The right figure shows that at ~0.5 second before SWD onset the wavelet energy is increased for all three channels. (**e**) Percentage of predicted and detected SWDs within the 4 hours recording of the first group of six WAG/Rij rats. Remember that no SWDs remained undetected by this (first) algorithm, the SWDs that were not predicted were quickly detected; Number of false positives across different states of alertness. For each rat and each state of alertness, 5 segments of 50 seconds duration were randomly selected in each recording for quantification of the number of false alarms.

The algorithm’s estimation of synchrony between the activity of brain structures is based on the analysis of both the synchronization of the electrical activity of neurons in the vicinity of a single electrode, *local synchronization*, and the synchronization between neuronal ensembles in cortex and thalamus, *global synchronization*. In two experiments we analyzed the multichannel cortical and thalamic EEG of genetically epileptic WAG/Rij rats. Within the framework of the algorithm, each EEG recording is considered as the macroscopic characteristic of the ensemble of interacting cells, located in the vicinity of the recording electrode. The obtained recordings were simultaneously processed with the help of a continuous wavelet transformation (see Methods) and the corresponding wavelet energies *W*_*i*_(*s*, *t*) were considered at every moment of time (200 times per sec). The analysis of the wavelet power spectra (Fig. 1b) of a single preictal EEG channnel (Fig. 1a) showed that early signs of synchrony, developing within each neural ensemble under consideration, occurred some seconds before SWD onset as represented by a local increase of the wavelet energy in the 5–10 Hz band. Along with local synchronization, interactions between the cortical and thalamic channel pairs increased and considering the power *W*(*s*, *t*)=Π*W*_*i*_(*s*, *t*) (Fig. 1c) an isolated pattern (circles in Fig. 1c) corresponding to preictal precursor activity was noticed. It showed that momentary changes in each EEG channel, taken for 4 (I) seconds and for ~0.5 seconds (II) before the onset of SWD indicated that an SWD was about to begin. This increase of wavelet energy took place in the 5–10 Hz frequency band (equivalent timescale 0.1–0.2 Hz^−1^, Δ*s*_1_), which implies that the spectral component of this EEG signal starts to synchronize between cortical layers and between cortex and thalamus.

The algorithm’s seizure prediction performance was evaluated with EEG recordings (four hours in duration) of WAG/Rij rats, a well validated genetic animal model of absence epilepsy as they experience several hundreds of spontaneously occuring SWDs per day. They are acompanied by mild facial myoclonus in an otherwise motionless animal^8^. It was found that the algorithm correctly predicted on average 88% of the SWD (range 80–100%), while the remaining SWDs were (early) detected (Fig. 1e). A high number of false positive predictions was noticed; they mainly occurred during light slow wave sleep, a state of alertness, in which neurons are slightly hyperpolarized and at high risk for seizure generation^9^. Only few false alarms were generated during active wakefulness, passive wakefulness and deep slow wave sleep (Fig. 1e).

**Figure 2:**
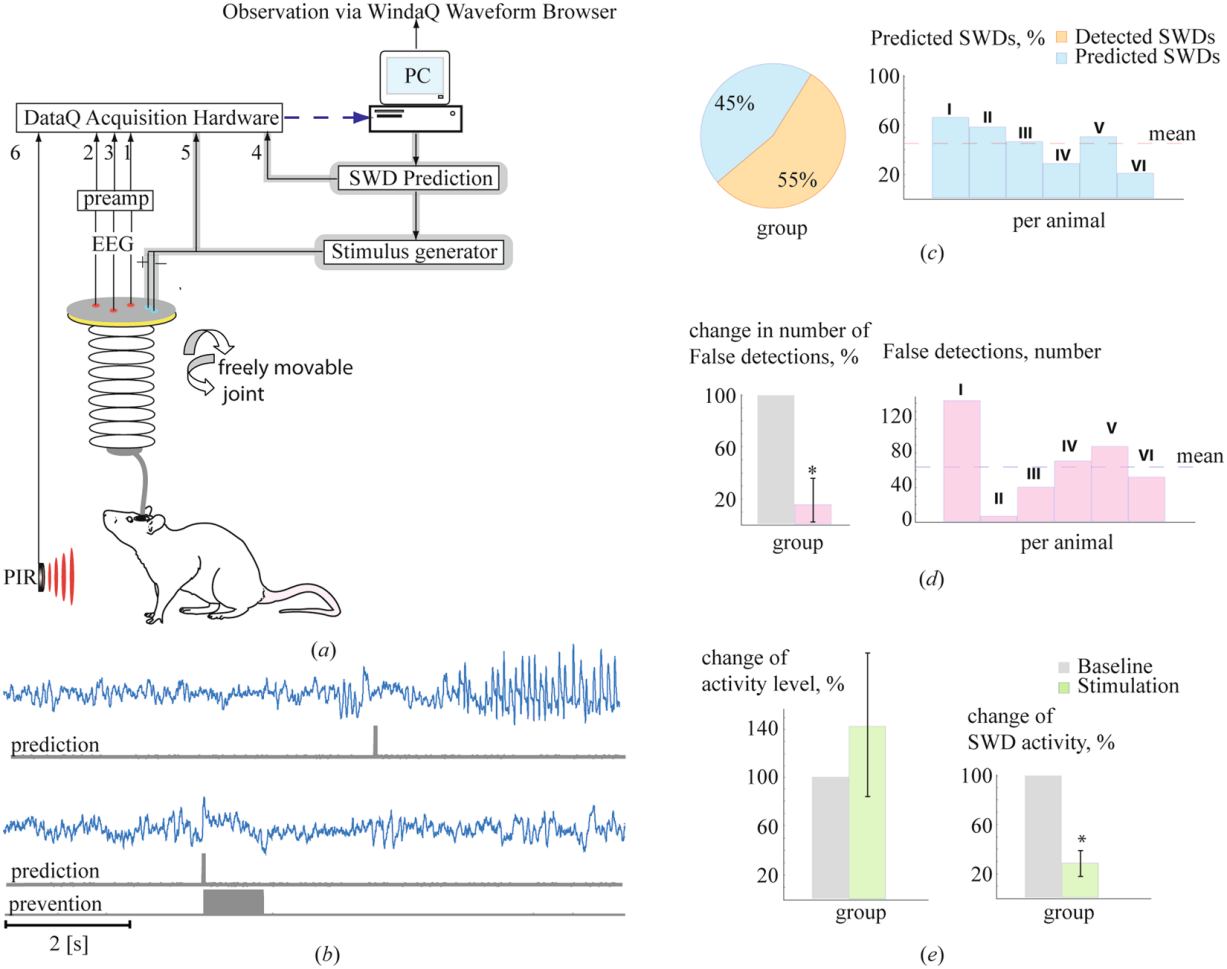
(**a**) The experimental setup of the brain-computer interface. The set of analog inputs 1–6 of the acquisition hardware correspond to the three EEG channels (1-3), a marker for predictions (4), the stimulation pulse train of 1 sec (5) and the signal from the passive infrared registration system (PIR) for movement detection (6), respectively. The dashed line corresponds to the digital input of the PC, the feedback is shown by the shadow. (**b**) The prediction (upper case) and prevention (lower case) of the SWD/absence seizure by delivering a pulse train of 1.0 second duration. (**c**) Mean percentage of correctly predicted and correctly detected SWDs by the algorithm including two additional critera (see para on “On-line precursor detection”). The data are from an one hour baseline recording of 6 WAG/Rij rats of experiment 2; (**d**) Number of false positives for each individual rat generated by the revised algorithm within the 1 hour baseline of the 6 WAG/Rij rats (right panel), and the relative decrease in the false positives rate (left pannel) between the first algorithm (gray) and the second algorithm (red). Note, to determine the relative decrease, one hour EEG recordings of rats of the first six WAG/Rij rats were analyzed by the algorithm without the two additional criteria and the false positive counts of this analysis were taken as 100% reference. (**e**) The total duration of the epileptic activity and the behavioral activity during the baseline and the stimulation session, averaged over the group of rats. The error bars show the standart error of the mean of the group.

The algorithm was extended in order to reduce the number of false positives. The extension was based on the simultaneous consideration of the wavelet energies of two other frequency bands, associated with synchronized brain activity: ${{\rm{\Delta }}}_{{s}_{2}}$(7–20 Hz, the range of sleep spindles, which in rats have a broad frequency spectrum), and ${\rm{\Delta }}{s}_{3}$ (the range of low-frequency oscillations (high (3–5) Hz delta, light slow wave sleep). The precursors were now automatically detected with the help of a set of logical conditions (for details see the Methods section and Supplementary Fig. 3). This resulted in a significant reduction of the false alarm rate of 83% (±3.3%) ((F(1,10) = 321.35, p < 0.001) (Fig. 2d), but with a trade-off in prediction sensitivity (Fig. 2d).

This latter version of the algorithm was subsequently implemented in a closed-loop deep-brain stimulation system. In this system the EEGs of freely moving WAG/Rij rats, recorded from two cortical and a thalamic site, were fed via an amplifier to a data acquisition system. The EEGs were analyzed in terms of synchrony in real time by the prediction algorithm. Whenever the level of synchrony exceeded a preset threshold value, and the two other criteria were met (see Methods sections for details), a marker was set in a free channel of the acquisition system and a constant current stimulator was triggered to deliver a low intensity 1 sec pulse train of 130 Hz to the rat (Fig. 2a,b). The preset detection threshold value was determined for each individual rat and varied between 0.10 and 0.40 (see section ‘EEG recording and processing’ for details regarding determination of the detection threshold).

We assumed that a pulse train of 130 Hz should prevent SWDs. This assumption was based on our previous work, in which it was established that this pulse train was rather effective (close to 90%) in interrupting onging SWDs^10^. A comparison of SWD activity between an one hour baseline recording in which no stimulation was applied, and SWD activity during an one hour stimulation session showed that SWD activity was reduced by 72 ± 10% (F(1,5) = 48.52, p < 0.001) (Fig. 2e). The reduction in SWD activity can be attributed to a combination of SWD prediction and prevention (in 45% of cases) and SWD detection and interruption. To support the conclusion that the reduction was not just the result of detection and disruption, we refer to the individual data of two rats in whom seizures were reduced by 98% and 100%, showing that total prevention of SWD activity by prediction and stimulation is feasable. In addition, inspection of corresponding wavelet energies, calculated for successful SWD prediction and prevention periods, depicted a strong momentary increase in wavelet energy within the 5–10 Hz band, signaling the development of an SWD in its preictal state and triggering the delivery of an electrical pulse train. The wavelet energy within this frequency band drastically dropped during and following 130 Hz stimulation, indicating that this electrical pulse train efficiently desynchronized the EEG and thereby successfully prevented the generation of a hypersynchronous SWD (Supplemantary Fig. 4).

In order to establish whether the remaining false positive detections, which also triggered the delivery of the electrical pulse train, might have affected the behaviour of the animals, we compared the activity of rats between baseline and stimulation hour. Behavioural activity was measured by a passive infrared registration (PIR) device. There was no difference in activity of the rats between the baseline and stimulation session (Fig. 2e) (F(1,5) = 0.476, p = 0.521), suggesting that the decrease in SWD activity induced by stimulation cannot be explained by an increase in behavioural activity. It is well known that motor activity precludes the occurrence of SWDs^8^. Furthermore, no other type of aberrant activity was observed in the EEG recordings of the animals during or after stimulation, and given the low intensity of electrical stimulation to prevent and disrupt SWDs and the relative short stimulation trains, we presume that this type of stimulation can be considered a relative safe intervention strategy.

The present research shows, that in contrast to the long- standing opinion that SWDs are unpredictable in nature^4^, SWDs can be predicted to a substantial degree and that a prediction algorithm can successfully be implemented in a brain computer interface that will greatly reduce SWD activity based on a combination of SWD prediction, prevention, detection and disruption. Previously, real-time, closed-loop DBS systems were aimed at interfering with ongoing SWDs as seen in models of childhood absence epilepsy but also in aquired epilepsy as in post-stroke models. The employed algorithms designed to detect SWDs used a variety of signal parameters such as line-length calculation, wavelet-filters or wavelet power in certain frequency bands, all reaching high sensitivity (and specificity) of SWD detection^11–16^. Likewise, on-line seizure detection algorithms for different types of epileptic seizures like in mesial temporal lobe epilepsy, are implemented in closed-loop DBS systems, in which local seizure activity can be quickly detected and interfered with by stimulation before seizure-related clinical signs become manifest in the animal or patient. Such algorithms currently reach high sensitivity and specificity of detection, despite high interindividual differences in seizure EEG parameters or interictal spiking^17, 18^. Different seizure prediction algorithms have been designed for seizures accompanying temporal lobe epilepsy, with different succes rates^19, 20^. For photosensitive patients, Kalitzin *et al*.^21^ as well as Parra *et al*.^22^ predicted absence as well as myoclonic seizures using a phase clustering index and reached a sensitivity of 85%. Their algorithm was not implemented and evaluated in an on-line setting, in contrast to the algorithm presented in the current paper.

The inclusion of additional sleep criteria intended to decrease the number of false alarms but reduced also the sensitivity of the prediction of SWDs from 88 to 45%. It needs to be pointed out that the same algorithm quickly detected all the remaining, unpredicted SWDs. In terms of SWD detection, this algorithm therefore still keeps a sensitivity of 100% for SWD prediction and early detection altogether. The inclusion of sleep criteria also reduced the number of false detections by 83%. We felt that especially in the light of stimulation safety (with the aim to interfere as often as necessary but as little as possible) such an inclusion is useful. The putative negative effects of interference by high frequency electrical stimulation of the brain was controlled by using behavioural activity as a read-out parameter, which did not significantly change between the baseline and stimulation session. In the long run, however, additional parameters like a combined video-EEG analysis, a thorough histological inspection of the stimulation site and analysis of sleep and wake states over longer (>24 h) stimulation sessions remain necessary for an accurate assessment of stimulation safety.

The high number of false positive predictions indicates that synchronization within and between brain structures is not unique for the generation of SWDs. It might be that the brain also ‘tries’ to generate SWDs at the periods indicated by false positive detections but fails to do so because another unknown requirement for SWD generation is not met. SWD preceding activity in the form of 5–9 Hz oscillations was earlier noticed in GAERS rats, another well characterized and validated genetic absence epilepsy model, rather similar to WAG/Rij rats. Similar to WAG/Rij rats, not all 5–9 Hz oscillations were followed by SWDs^23, 24^.

Both the sensitivity and specificity of our SWD prediction algorithm might be improved by a solid selection of the most optimal combination of recording sites, in combination with a refinement of the ways in which the brain can be stimulated to prevent the occurrence of SWDs. In addition, a thorough investigation of seizure transition periods at the level of single and multi-unit activity in cortex and thalamus might reveal additional parameters that will be useful for the improvement of SWD prediction. A consistent change in firing activity of the somatosensory thalamus was observed to precede SWD in a mouse model with a region-specific knockout of Scn8a^25^. In the latter work, however, the change in firing activity was not employed to predict SWD.

The current work is, to the best of our knowledge, the first which combines the separate disciplines of seizure prediction and closed loop deep brain stimulation in the domain of absence epilepsy. The ideas presented here might also be useful for the development and refinement of sensitive and selective BCI seizure prediction/prevention systems for other types of generalized epilepsies.

## Methods

### Rats

Male 6–7 months WAG/Rij rats (body weight ca. 350 gr) served as experimental subjects. The rats were born and raised in the Department of Biological Psychology, Donders Centre for Cognition, of Radboud University, Nijmegen, The Netherlands. Before surgery they were housed in pairs, after surgery they were housed individually (High Makrolon cages with Enviro Dri bedding material and cage enrichment). Rats were kept on a 12:12 light cycle (lights off phase between 8.30 and 20.30 h), with food and water ad libitum. All efforts were made to restrict the number of rats and to limit discomfort. All procedures and protocols were carried out in accordance with the guidelines of the council of the European Union of 22 September 2010 (2010/63/EU) and approved by the Ethical Committee on Animal Experimentation of Radboud University, Nijmegen (RU-DEC).

### Surgery

The stereotactic surgery was performed under isoflurane anesthesia. The first group of 6 WAG/Rij rats was implanted with a custom-made electrode set. The tips of two electrodes were aimed at the deep layers of the somatosensory cortex, a third electrode was aimed at the posterior nucleus of the thalamus. The electrodes consisted of stainless steel wires with a diameter of 0.2 mm, insolated with polyimide. Only the tip of each electrode wire was not insolated. Cortical locations of the tip of the EEG recording electrodes were (A/P = 0, M/L = −4.6 mm, H = −4.1; A/P = 0, M/L - 4.6 mm, H = 4.6) and for the thalamus (A/P: −3.6, M/L: −2, H: 6.4 mm), ground and reference electrodes were placed on top of the cerebellum.

A second group of six rats was implanted with a tripolar (MS 333/2a, Plastic One, Roanoke, VA, USA) and two bipolar sets of electrode (MS 303/11, Plastic One). The first electrode set was used to record a thalamic EEG (A/P: −3.6, M/L: −2.4, H: 6.4 mm), with a reference and earth electrode both above the cerebellum. The first bipolar set of electrodes, also used for recording, was aimed at the depth of the somatosensory cortex (A/P: both electrodes −0.5, M/L: 4.5 and 5.0, H: 4.5 and 5.0 mm), the second set of electrodes consisted of a pair of bipolar stimulation electrodes, covering the focal region with the tips aimed at the deep somatosensory cortex or the underlying white matter (coordinates A/P: 2.0, M/L: 4.4, H: 4.1 mm and A/P: −3.0, M/L: 4.8, H: 2.95 mm). All coordinates were derived from the stereotactic atlas of Paxinos and Watson^26^.

All electrode sets were fixed to the skull with dental cement (Simplex Rapid, Kemdent, Purton, Swindon, Wiltsher, UK). Prior to surgery, the animals received an injection of atropine (0.05 ml intramuscular) and rimadyl (5 mg/kg subcutaneous). They also received rimadyl 24 and 48 hours after surgery (5 mg/kg subcutaneous). Rats were allowed 14 days to recover before any electrophysiological experiments were performed.

### EEG Recording and Processing

Rats were habituated to the Plexiglas recording cage (20 × 35 × 25 cm) and cables for 16 hours: the leads were attached to a swivel-contact to allow registration and stimulation in the freely moving animals. EEG signals were passed throuth a physiological amplifier (TD 90087, Radboud University, Nijmegen, Electronic Research Group) and a band pass filter with cut-off points at 1(HP) and 100(LP) and a 50 Hz Notch filter. Differential EEG recordings were obtained from the two cortical sites and the postero/lateral thalamus. The EEG signals were digitized by a WINDAQ-recording-system (DATAQ-Instruments Inc., Akron, OH, USA) with a constant sample rate of 500 Hz. The PIR detector registered the movements of the rat (RK2000DPC LuNAR PR Ceiling Mount, Rokonet RISCO Group S.A., Drogenbos, BE). EEGs and behaviour of the first group of 6 rats were recorded for 4 hours during the dark phase. These recordings were analyzed by the first version of the algorithm (without the two sleep criteria). EEGs and behaviour of the second group of 6 rats were first recorded between 9.00 and 16.00 h (baseline). For each rat one hour of this baseline was analyzed several times by an off-line version of the algorithm with the two additional criteria: each time a different detection threshold value was explored in order to identify an individualized and ‘optimized’ precursor detection threshold value, which was later used in the ‘online seizure prediction’ experiment. This ‘optimal threshold’ was chosen based on a comparison between the number of correctly detected SWDs, the number of missed SWDs, and the number of false positive detections revealed for the different threshold values tested.

In addition to the precursor detection threshold, the threshold for electrical cortical stimulation for SWD interruption was also determined for each individual rat by finding the intensity at which three subsequent SWDs were aborted by a 1 sec 130 Hz pulse train. This intensity was used in the second experiment. On the prediction/stimulation day of this experiment, one hour base-line EEG recording was followed by 1 hr SWD prediction and electrical stimulation recording session.

### Histology

The location of the electrodes used in the second experiment were histologically verified upon completion of the experiment^26^. A direct-current (9 V, 15 s duration) was first passed through each electrode in the anaesthetized rat. Next, rats were perfused with a potassium-ferrocyanide-formaldehyde-phosphate solution; brains were removed and post-fixed in a 30% sucrose solution, in 0.1 ml PBS. Brains were cut in 40 μm coronal slices to determine electrode locations. Animals with a confirmed electrode tip in the somatosensory cortex or the underlying white matter were included in the statistical analyses (Supplementary Fig. 1). Positions of the electrode tips from rats used in the first experiment were previously published^12^.

### Wavelet Analysis

The time-frequency decomposition of each EEG signal *X*_*i*_(*t*) was performed with a continuous wavelet transform^27^:

1 $${A}_{i}(s,t)=\frac{1}{\sqrt{s}}{\int }_{t-s}^{t+s}{X}_{i}(t^{\prime} ){\phi }^{\ast }(\frac{t-t^{\prime} }{s})dt^{\prime} $$

with the specially designed mother complex function:

2 $$\phi (\eta )={\pi }^{1/4}\mathrm{Exp}[i{\omega }_{0}\eta ]\mathrm{Exp}[\frac{-10{\eta }^{4}}{2}],$$

where *s* = 1/*f* is the timescale [in s = Hz^−1^], *ω*_0_ = 2*π* is the parameter of the wavelet function^16^, *f* is the linear frequency [in Hz]. The used mother wavelet function (2) is a modification of the well-known Morlet wavelet^27^ which is characterized by a better localization in time in comparison to the standard Morlet wavelet and, therefore better suited for on-line usage and detection of short phasic acticity due to a better temporal resolution of local peculiarities in the EEG signal^28^.

According to Eq. (1) the wavelet coefficient *W*(f*, t*) is calculated for a certain frequency f* (or timescale s* = 1/f*) and current moment of time t* by integrating over the time window, the duration of which *δ* is defined by f*. In this case the analyzed moment of time is the middle of the window which, in turn, causes a delay in online detection. The dependency of the window length on the value of frequency belonging to the analyzed frequency bands [3–20 Hz] of interest is shown both for Morlet wavelet *δ*_1_ and modified wavelet *δ*_2_ in Supplementary Fig. 2. It can be seen that the usage of the modified wavelet shortens the duration of the window and, therefore the delay. It can also be seen that the maximal window size, used for the calculation of the wavelet spectrum, is about 600 ms, the wavelet function is shown for both the Morlet wavelet and modified wavelet. The delay is shown to be reduced from 1.2 seconds to 0.3 seconds which appears fast enough to on-line detect SWD precursor patterns.

The power spectrum ${W}_{i}(s,t)={A}_{i}^{2}(s,t)$ was calculated for the frequency range *f* ∈ [3, 20] Hz by using the proposed wavelet. The algorithm was implemented in Borland Delphi 7 using the DATAQ ActiveX Control Library and run on a Personal Computer (Intel Core2Quad, 4.0 Gb RAM, Win 7,64). This hardware configuration allowed us to perform the wavelet transformation in real time with the time step Δ*t* = 5 × 10^−3^ seconds (200 Hz) – small enough to provide high-quality signal decomposition in the frequency range under consideration. The resulting measure of wavelet spectra of the multichannel EEG, *W*(*s*, *t*) was defined as the product of the spectra obtained for all EEG recordings of the set *W*(*s*) = *W*_*1*_(*s*) × *W*_2_(*s*) × *W*_3_(*s*) at every moment in time. Subscript 1, 2 and 3 represented two subgranual cortical signals from the somatosensory cortex and one from the thalamic Posterior (PO) Nucleus, respectively. The values *W*_Δ*sj*_(*t*), corresponding to the spectral energy of the timescales ${\rm{\Delta }}{s}_{j}$, were derived with the equation:

3 $${W}_{{\rm{\Delta }}{s}_{j}}(t)=\frac{1}{{\rm{\Delta }}{s}_{j}}{\int }_{s\in {\rm{\Delta }}{s}_{j}}\frac{1}{\tau }{\int }_{{t}_{0}=t-\tau }^{t}W(s,{t}_{0})dsd{t}_{0,}j=\overline{1,3,}$$

where the integration was performed both over the range of time scales and the time interval *τ* = 500 ms which was determined experimentally taking into account the minimal duration of the precursor.

### On-line precursor detection

The first step for the automatic recognition of precursor was to determine the wavelet energy ${W}_{{\rm{\Delta }}{s}_{1}}(t)$ in the 5–10 Hz band. It was found that the value of ${W}_{{\rm{\Delta }}{s}_{1}}(t)$ increased before the onset of SWDs. The value of ${W}_{{\rm{\Delta }}{s}_{1}}(t)$ was compared to the threshold value *W*_*th*_, which was determined for each individual rat by a preliminary analysis of the wavelet energy during the preictal state and different types of interictal (=background) activity. This allowed us to choose a threshold value which was higher than the energy of the background activity but smaller than the energy of the preictal precursor activity. Thus, precursor activity of SWDs were detected via the condition ${W}_{{\rm{\Delta }}{s}_{1}}(t) > {W}_{th}$. It can be seen in Fig. 1 that this condition was responsible for a large number of false alarms during slow wave sleep due to an increase in synchronization between the different channels in sleep and sleep spindle related frequency bands. In order to reduce the number of false alarms caused by any type of synchronized neuronal activity, additional ‘sleep’ criteria were introduced. These criteria included the simultaneous consideration of two other frequency bands, or timescales, corresponding to common patterns of synchronic neural activity: ${\rm{\Delta }}{s}_{2}$ (the range of sleep spindles: 7–20 Hz) and ${\rm{\Delta }}{s}_{3}$ (the range of low-frequency oscillations (delta-precursors): 3–5 Hz characterizing light slow wave sleep)^7^. For these ranges the values of mean energy ${W}_{{\rm{\Delta }}{s}_{1}}(t)$ was calculated by averaging *W*(*s*, *t*) over the range of the time scales ${\rm{\Delta }}{s}_{j}$ and an interval of *τ* = 0.5 seconds (See Methods, Eq. (3)).

The three rectangular windows in Supplement. Fig. 3b, correspond to the areas of values on the plane (*s*, *t*) over which the ${W}_{{\rm{\Delta }}{s}_{1,2,3}}$ were calculated at time *t* = t*, each of duration *τ* and width ${\rm{\Delta }}{s}_{1}$, ${\rm{\Delta }}{s}_{2}$, ${\rm{\Delta }}{s}_{3}$, shown by the solid, dotted and dashed lines respectively. During the online calculation the current moment of time *t* was located at the right-hand side of a rectangle. Thus, the algorithm stored 0.5 s of prior signals and used it for averaging. When new data arrived from the hardware, the rectangles shifted to the right with stepsize $\delta t=\frac{1}{{s}_{R}},$ where *s*_*R*_ = 200 Hz (the sample rate). The quantities ${W}_{{\rm{\Delta }}{s}_{i}}$ were then calculated for the next moment of time. One can see that the value of the mean energy ${W}_{{\rm{\Delta }}{s}_{1}}$, obtained during the presence of precursor activity became larger than ${W}_{{\rm{\Delta }}{s}_{2,3}}$ and, moreover, significantly exceeded the same value corresponding to the background activity. Therfore, using the threshold value *W*_*th*_ one can automatically detect the precursor with the help of the previous and two additional conditions: (i) ${W}_{{\rm{\Delta }}{s}_{1}}(t) > {W}_{th}$, (ii) ${W}_{{\rm{\Delta }}{s}_{1}}(t) > {W}_{{\rm{\Delta }}{s}_{2}}(t)$, and (iii) ${W}_{{\rm{\Delta }}{s}_{1}}(t) > {W}_{{\rm{\Delta }}{s}_{3}}(t)$. Conditions (ii) and (iii) were used to distinguish the precursor events from sleep spindles and low-frequency delta activity. Like the SWDs, these types of EEG activities were also accompanied by synchronization within the deep layers of the cortex and between cortex and thalamus.

The on-line SWD prediction performace of the refined algorithm (sensitivity and specificity) was assessed by measuring the number of correctly predicted SWDs, the number of missed SWDs, the number of detected SWDs, and the number of false positive detections, during the 1-hour baseline recording preceding the stimulation session, during which the algorithm analyzed the recorded EEG in real-rime. Sensitivity of the algorithm was calculated as (number of correct predictions/(number of correct predictions + number of missed SWDs + number of detected SWDs)) * 100% and reached a value of 45%. Specificity of the algorithm was calculated as (number of correct predictions/(number of correct predictions + number of false positives)) * 100% and reached a value of 24%. An SWD was defined as correctly predicted when it occurred maximally 1 second following the marker indicating the detection of a precursor. The onset of an SWD was defined as the first epileptic spike (sharp spike of at least twice the amplitude of the background LFPs) which is visible in all cortical and thalamic recording channels and is followed by rhythmic SWD activity, as in previous work of our group^5, 29^.

### Stimulation

The stimulus generator was controlled by custom-made software, that allowed for the implementation of a set of stimulation pulse parameters (e.g. duration, frequency, pulse width, intensity) from a text file. In order to minimize idle time once a precursor was detected, the prediction algorithm immediately activated the stimulator via a hardwire connection thread and a precursor detection marker was sent to the hardware of the data acquisition system. This hardwire connection thread, responsible for the communication between the prediction algorithm and the stimulus generator, was implemented via the parallel-printer port of the computer. It was activated for 1.0 s and simultaneously blocked any signals arriving from the prediction algorithm, in order to prevent delivery of additional stimulations caused by artifacts that may appear in the cortical EEG during the delivery of a pulse train. The precursor detection marker was a 0.05 s pulse.

## Electronic supplementary material

supplementary figures
