# Supplementary material for: Absence Seizure Control by a Brain Computer Interface
Source: Sci Rep. 2017 May 29;7:2487. doi: 10.1038/s41598-017-02626-y (PMC5447660; doi:10.1038/s41598-017-02626-y)
Supplement: Supplementary file 1 — supplementary figures [file 41598_2017_2626_MOESM1_ESM.pdf]

Absence Seizure Control by a Brain Computer Interface

Vladimir A. Maksimenko, Sabrina van Heukelum, Vladimir V. Makarov, Janita Kelderhuis,
Annika Lüttjohann, Alexey A. Koronovskii, Alexander E. Hramov, Gilles van Luijtelaar

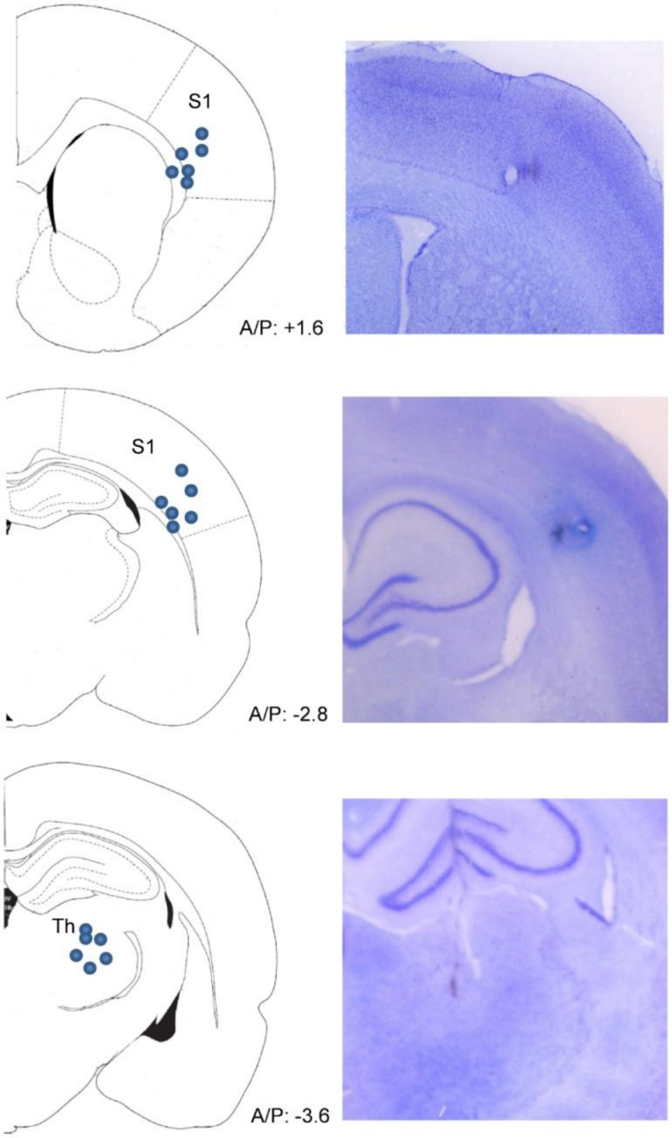

Supplementary Fig. 1. Histological verification of stimulation electrode location in the somatosensory cortex (S1) and a recording electrode in postero/lateral thalamus (Th). Left panel of the figure is adapted from Paxinos and Watson (2008) [26].

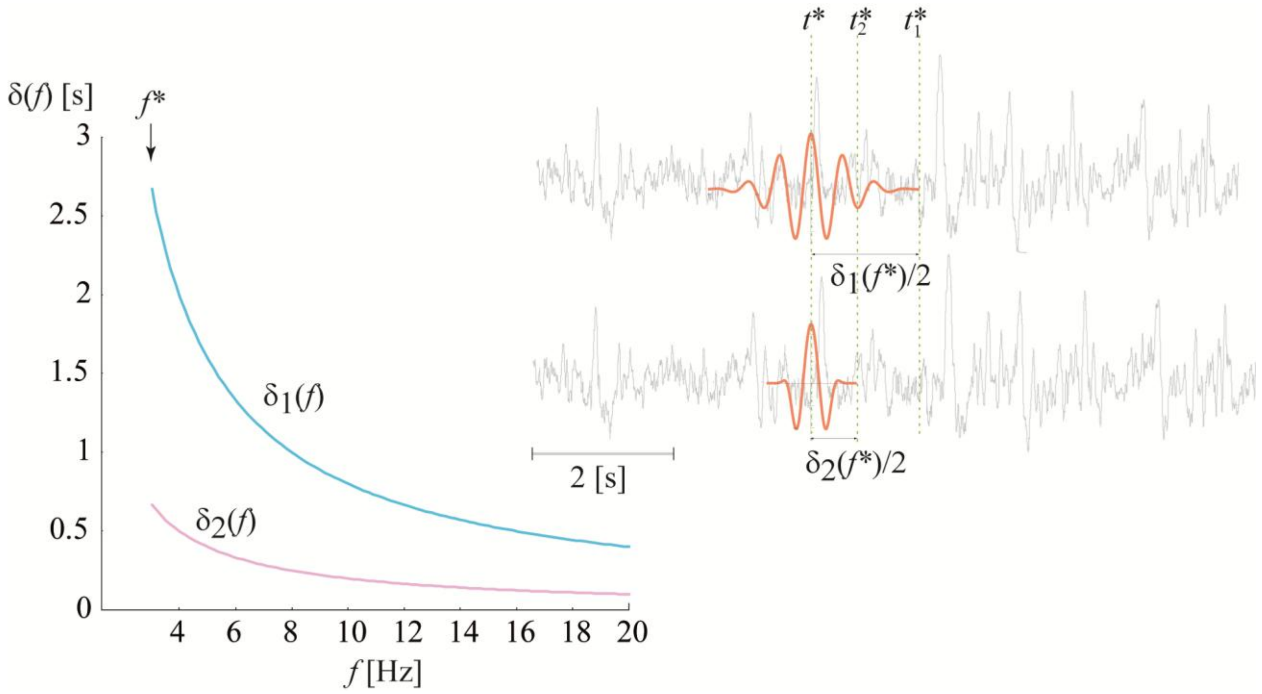

Supplementary Fig. 2. (Left panel) The dependence of the window length δ_1 and δ_2 on the frequency within the analyzed frequency band [3-20 Hz] for the Morlet and modified wavelet respectively. (Right panel) Illustration of the calculation of the wavelet coefficient for *f**=3 Hz (largest window length) with the help of Morlet wavelet (top) and modified wavelet (bottom).

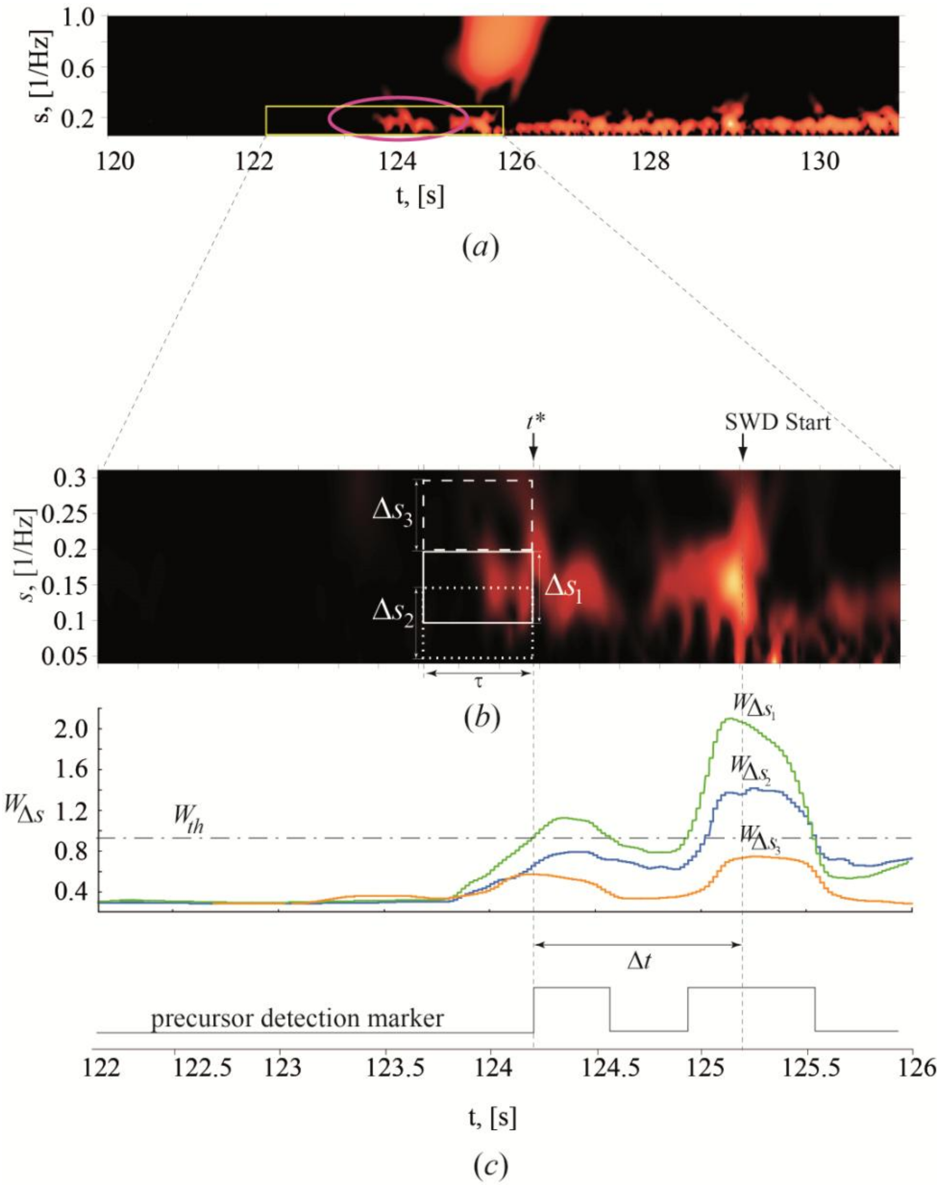

Supplementary Fig. 3. (*a*) the wavelet energy spectrum *W*(*s,t*), illustrated here for an interval corresponding to the transition from normal activity to the onset of SWD (at 125.2). The by the oval marked pattern represent SWD precursor activity. (*b*) Detailed (from the rectangle in panel *a*) illustration of *W*(*s,t*) inter- and preictal. The rectangular windows in panel (*b*) represent the different time scales (frequency bands): Δs_1 - (solid line, 5-10 Hz, (0.2-0.1)/Hz)), Δs_2 - (dashed line 7-20 Hz, (0.15-0.05)/Hz)), and Δs_3-

(dotted line 3-5 Hz, (0.3-0.2)/Hz)) and interval τ, for which the value of $W(s,t)$ is averaged. (*c*) $W(s,t)$ is the product of the three values of $W_{\Delta s_i}$ at every moment of time, averaged over time interval τ. W_{th} is the threshold energy value, used for prediction. Δt— time between the detected precursor activity and the onset of a SWD.

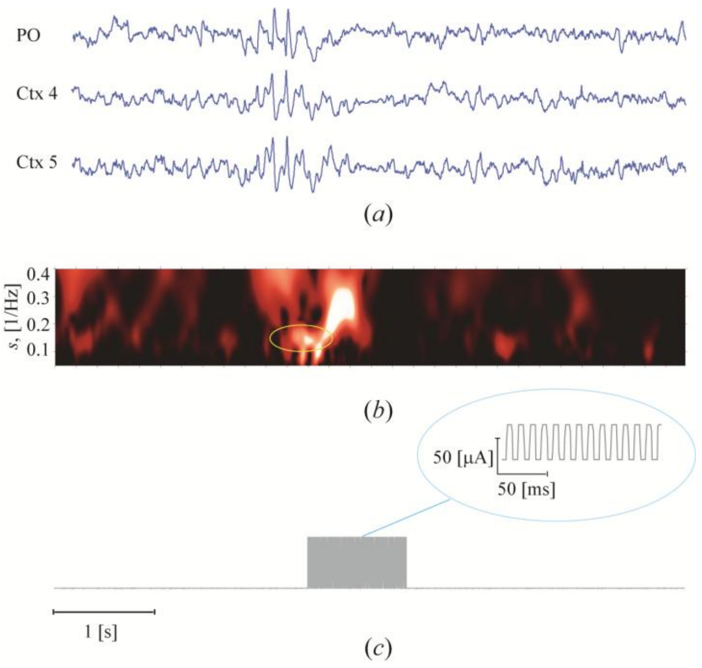

Supplementary Fig. 4. SWD prevention by means of electrical stimulation with 1 sec pulse train of 130 Hz and 0.4 ms pulse duration. EEG signals, taken from postero/lateral thalamus (PO) and cortex layer 4 and 5 (*a*), the distributions of the wavelet energy $|W(s,t)|$ (b) and the pulse train (c) (the structure of the pulse is shown in detail).
